# Antibiotic utilization at an orthopedic inpatient department in a large governmental hospital in the north of the West Bank, Palestine; a retrospective observational study

**DOI:** 10.1186/s12879-024-09686-2

**Published:** 2024-08-22

**Authors:** Raed Masalma, Ahmad Ghanim, Mahmoud Jarrar, Thabet Zidan, Abdulsalam Alkaiyat, Mazen Abdalla, Mohammad M. Jaber, Ismail Qattawi, Nagham Joudeh, Rasha Khayyat

**Affiliations:** 1 Present Address: Department of Medicine, Faculty of Medicine and Health Sciences, An-Najah National University, Nablus, 44839 Palestine; 2 Department of Public Health, Faculty of Medicine and Health Sciences, An-Najah National University, Nablus, 44839 Palestine; 3 Department of Orthopedic Surgery, An-Najah National University Hospital, Nablus, 44839 Palestine; 4 Rafidia Surgical Hospital, Nablus, Palestine; 5 Department of Biomedical Sciences, Faculty of Medicine and Health Sciences, An-Najah National University, New Campus, Building: 27, Office: 2140, P.O. Box 7, Nablus, 44839 Palestine

**Keywords:** Antibiotics, Antibiotic resistance, Antibiotic stewardship, Orthopedic, Surgical site infections, Antibiotic prophylaxis, ATC/DDD, DU90%, AWaRe policy

## Abstract

**Background:**

Studies evaluating the patterns of antibiotic consumption are becoming increasingly necessary as a result of the increased use of antibiotics and development of antibiotic resistance globally. This study aimed to evaluate the use of antibiotics in in terms of both quantity and quality at the largest surgical hospital in the north of the West Bank, Palestine.

**Methods:**

An observational retrospective study with a total population sampling method was conducted to collect data from the inpatients of the orthopedic departments of a large governmental hospital in the northern West Bank, Palestine. The data were collected from patients’ files and evaluated using the anatomical therapeutic chemical and defined daily dose (ATC/DDD) methodology, and the drug utilization 90% (DU90%) index. The ATC/DDD methodology, designed by the World Health Organization (WHO), as a well-trusted and standardized tool that allows measuring and comparing antibiotic utilization across different contexts. Antibiotic prescriptions were classified using the World Health Organization Access, Watch and Reserve classification (WHO AWaRe).

**Results:**

Of the 896 patients who were admitted to the hospital in the year 2020 and included in the study, 61.9% were males, and 38.1% were females. The percentage of patients who received antibiotics was 97.0%, and the overall antibiotic usage was 107.91 DDD/100 bed days. The most commonly prescribed antibiotic was cefazolin (50.30 DDD/100 bed days), followed by gentamicin (24.15 DDD/100 bed days) and ceftriaxone (17.35 DDD/100 bed days). The DU90% segment comprised four different agents. Classification of antibiotics according to the WHO AWaRe policy revealed that 75.9% of antibiotics were prescribed from the access list.

**Conclusion:**

This study comes as part of the efforts exerted to combat the growing problem of antibiotic resistance in Palestine. Our results showed that the consumption of antibacterial agents in the orthopedic unit at a large governmental hospital in Palestine was relatively high. The results of this study provide valuable insights for the decision-makers to create policies aimed at regulating antibiotic prescriptions. This study also aims to provide a look into the antibiotic prescription patterns, offering a clearer understanding of the current situation of antibiotic consumption in Palestine. It also emphasizes the need for antibiotic stewardship and surveillance programs.

## Background

Antibiotics are an essential class of pharmacological agents utilized to combat bacterial infections and thus play a crucial role in modern medical practice [1]. Arising from natural sources or being created synthetically in laboratories, antibiotics have been shown to have the power to impede the proliferation and growth of bacteria. Additionally, they have the ability to kill these microorganisms, effectively halting infectious processes [2]. Long-term and improper antibiotic use, whether in medical settings, nonprescription contexts, or livestock and poultry fields, has influenced the evolution of bacterial strains with resistance mechanisms [3–5].

Antibiotics are necessary for both prophylactic and therapeutic purposes in the field of surgery. The goal of prophylactic antibiotic therapy is to reduce the incidence of surgical site infections (SSIs), leading to a reduced hospital stay and improved outcomes [6]. The field of orthopedic surgery is not exempt from this problem, as resistant strains may impair the effectiveness of well-established treatment regimens, thereby resulting in treatment failure and increased healthcare expenses [7, 8].

Guidelines published by medical societies and public health authorities are essential tools for promoting the proper use of antibiotics. To achieve a balance between efficacy and reducing the risk of antibiotic resistance, these guidelines outline recommendations for antibiotic selection, dose, time of administration, and duration of therapy [9, 10]. Antibiotics are divided into Access, Watch, and Reserve groups according to the WHO’s AWaRe classification, which was designed in 2017 to encourage proper usage and combat antimicrobial resistance. “Access” refers to antibiotics with lower resistance potential, used for common infections. “Watch” refers to those with higher resistance potential and limited use, while “Reserve” refers to last-resort antibiotics used for multidrug-resistant infections [11].

One study in Palestine was conducted in the North of the West Bank, where information from 400 orthopedic, abdominal, and gynecologic procedures performed in 2011 was gathered. It concluded that the lack of guidelines in Palestine may be the cause of the low adherence to proper surgical prophylaxis in all departments [12].

This study’s objective was to analyze the patterns of antibiotic usage at the largest surgical hospital in the West Bank’s northern region, and compare them with those of international counterparts. This goal was achieved by assessing antibiotic utilization using the Anatomical Therapeutic Chemical and Defined Daily Dose (ATC/DDD) methodology [13], the drug utilization 90% (DU90%) index [14], and the WHO’s AWaRe classification. We chose to implement the ATC/DDD methodology in our study because it enables the collection of quantifiable and standardized data on antibiotic consumption, facilitating comparisons with other international, national, and regional facilities. Therefore, we could use the emerging data from our study to support the implementation of policies aimed at guarding the use of antibiotics and improving quality outcomes.

## Methodology

### Study design and settings

This observational and retrospective study was conducted in one of the largest surgical hospitals in Palestine to evaluate antibiotic utilization from January 1st to December 31st, 2020. The hospital is considered a secondary hospital and has a capacity of 200 beds, it’s located in the city of Nablus in the North of the West Bank, and provides its services to the public.

### Study population and data collection

All adult patients admitted to the orthopedic ward at the hospital who stayed for at least one night during the study period were included in our study population. All patients under the age of 18 were excluded from the study, since the ATC/DDD methodology used in the study applies only to the adult population [13].

We have developed a data abstraction template along with accompanying instructions. Trained and regulated individuals were responsible for abstracting data. We conducted a thorough examination and randomly verified the acquired data to ensure its validity and reliability. The data were collected from the patients’ files found in the hospital archives, and the charts of 1909 patients were reviewed to collect the relevant data; A total of 896 patients met the inclusion criteria and were included in the study.

The collected data included the age and gender of the patients, their admission and discharge date, their diagnosis on admission, the number of surgeries performed and their outcomes. Information about the antibiotics administered to patients, such as the name, dose, frequency, duration of treatment, and route of administration, was also collected.

### Study tool and variables

The aim of this study was to measure antibiotic utilization, both from quantity and quality aspects. To achieve this aim, three types of methodologies and measurement tools were used, which included:

- The ATC/DDD methodology was used to assess antibiotic utilization quantitatively and involved linking each drug to its corresponding ATC code and DDD. This approach standardized drug measurements and provided a consistent metric for global, local, and interfacility comparisons. The DDD, defined as the assumed average daily maintenance dose for a drug’s primary adult indication, was assigned uniquely to each ATC code and route of administration. Antibiotic consumption data were converted from grams (g) to DDDs using the 2023 ATC/DDD index and expressed as DDD/100 bed days, which is an important indicator for in-hospital antibiotic use.
  DDD/100 bed days = Number of units administered in a given period (milligram) ×100 /DDD (milligram) × number of bed days.
  Bed day: a day during which a person is confined to a bed and in which the patient stays overnight in a hospital [13].
- The DU90% index was utilized to assess the quality of antibiotic utilization, categorize antibiotics based on their DDD prescription quantities. This tool identified the antibiotics that constituted 90% of the total consumption, with the premise that prescribing fewer agents signifies improved prescription patterns. This assessment aligns with the goal of optimizing antibiotic use [14].
- The WHO’s AWaRe classification was also used to assess the quality of antibiotic utilization and categorized antibiotics into three groups: access, watch, and reserve. This classification aimed to ensure that at least 60% of antibiotic consumption falls within the “access” category, emphasizing the importance of responsible antibiotic utilization in healthcare settings [11].

### Statistical analysis

The researcher individually coded the collected data, after which it was entered and subjected to analysis using the Statistical Package for Social Sciences (SPSS) version 28.0 for Windows. Continuous variables were examined through descriptive statistics, which included calculations of means, medians, interquartile range (IQR), and standard deviations, whereas discrete variables were subjected to frequency analysis.

### Ethical approval and consent to participate

Ethical approval was obtained from the hospital administration, ethics, and/or research committees. Additionally, approval was obtained from the Institutional Review Boards (IRB) of An-Najah National University and the Ministry of Health before the start of the study.

Formal written informed consent was waived since there was no direct patient contact and this was a quality assurance study aimed at improving care. All collected data remained confidential and were used only for the purpose of this study and patients’ names were anonymized.

## Results

### Demographic and clinical characteristics

A total of 896 patients were included in our study. In general, the mean age was 50.97 (SD = 20.912) years, with the largest category being above 60 years and containing 323 patients (36.0%). A total of 555 (61.9%) patients were males, and 401 (44.8%) had at least one chronic disease. The most common chronic disease was hypertension (30.4%), followed by diabetes mellitus (22.1%), chronic heart disease (9.4%), and rheumatic diseases (5.6%). Regarding the number of operations that patients underwent during their hospital stay, 770 (85.9%) of them had at least one operation, and only 1.5% of the patients had more than one surgery.

The median length of hospital stay was 3 with an IQR of (2–5) days, ranging from 1 to 43 days. Regarding patient outcome, 852 (95.1%) patients were discharged from the hospital.

A total of 869 (97.0%) patients received antibiotics during their stay, 417 (46.5%) received only one antibiotic, 369 (41.2%) received only two antibiotic drugs, and 83 (9.3%) received more than two antibiotics. Table 1 presents the general characteristics of the patients.

**Table 1:** Participants’ demographic and clinical characteristics (*n* = 896)

Variable	*n*(%)
Gender	
Male	555(61.9)
Female	341(38.1)
Age (years)	
18–30	206 (23.0)
31–45	167 (18.6)
45–60	200 (22.3)
> 60	323 (36.0)
Chronic diseases *	
Yes	401 (44.8)
No	495 (55.2)
Operated	
Yes	770 (85.9)
No	126 (14.1)
Prescribed antibiotics	
Yes	869 (97.0)
No	27 (3.0)
Performed culture and susceptibility tests	
Yes	64 (7.1)
No	832 (92.9)
Outcome	
Discharged from the hospital	852 (95.1)
Shifted within the hospital to another department	0 (0.0)
Discharged on request	24 (2.7)
Referred to another hospital for further treatment	17 (1.9)
Died	3 (0.3)
	**Median (IQR)**
Length of hospital stay (days)	3.00 (2.00–5.00)

Abbreviations: n, number of inpatients, IQR, interquartile range

*Chronic diseases included: Diabetes, Hypertension, Chronic Heart disease, Chronic Respiratory diseases, Chronic GI diseases, chronic kidney disease, Neurologic Diseases, Malignancy, Rheumatic diseases, Psychiatric disorders

### Diagnoses and indications for admission

The most common diagnosis was fractures of the spine and limbs with a total of 504 (56.2%) patients, and 486 (96.4%) of those patients received antibiotics during their stay at the hospital. Femur fractures were the most common fracture diagnosis and accounted for 255 (28.5%) of the total cases and lower leg fractures accounted for 145 (16.1%) of the total cases. Infectious diagnoses accounted for 65 (7.3%) of all the cases. Osteoarthritis and disorders of the synovium and tendons accounted for 101 (11.3%) and 94 (10.5%) respectively. Table 2 describes the numbers of inpatients who were prescribed antibiotics with respect to the most common diagnoses at the orthopedic department.

**Table 2:** Numbers of inpatients who were prescribed antibiotics with respect to most common diagnoses

ICD-10 Codes and Diagnoses	Frequency of diagnosis	Inpatients prescribed antibiotics
	*n*(%)	n*(%)
S 32-S 82 Fractures of spine and limbs	504 (56.2)	486 (96.4)
S 32 lumbar spine and pelvis	6 (0.7)	5 (83.3)
S 42 shoulder and upper arm	38 (4.2)	38 (100.0)
S 52 forearm	41 (4.6)	41 (100.0)
S 62 wrist and hand level	19 (2.1)	18 (94.7)
S 72 femur	255 (28.5)	242 (94.9)
S 82 lower leg, including ankle	145 (16.1)	142 (97.9)
Multiple fractures	5 (0.6)	5 (100.0)
M15-M19 Osteoarthritis	101 (11.3)	100 (99.0)
M65-M67 Disorders of synovium and tendon	94 (10.5)	92 (97.9)
All bacterial infectious diagnoses	65 (7.3)	65 (100.0)
Other diagnoses	127 (14.1)	121 (95.3)

The percentage n* (%) is calculated for the number of inpatients who were prescribed antibiotics with a specific diagnosis out of the total number of inpatients with that diagnosis. Abbreviations: ICD, International Classification of Diseases

### Antibiotic consumption

The overall antibiotic use was 107.91 DDD/100 bed days. A total of 17 antibiotic drugs were used. The most commonly prescribed antibiotic was cefazolin (50.3 DDD/100 bed days), followed by gentamicin (24.1 DDD/100 bed days), ceftriaxone (17.4 DDD/100 bed days), and vancomycin (6.3 DDD/100 bed days). Cefazolin was given to 785 (87.6%) patients, while gentamicin was administered to 354 (39.5%) patients. Table 3 details the frequency and consumption antimicrobial prescriptions.

**Table 3:** Antimicrobials prescribed for patients and antimicrobial consumption (DDD/100 bed days)

Antimicrobial agent	*n*(%)	DDD/100 bed days
First-generation cephalosporins (Cefazolin)	785 (87.6)	50.30
Second-generation cephalosporins (Cefuroxime)	18 (2.0)	1.22
Third-generation cephalosporins		
Ceftriaxone	180 (20.1)	17.35
Ceftazidime	4 (0.4)	0.08
Aminoglycosides (Gentamicin)	354 (39.5)	24.15
Penicillins		
Amoxicillin-clavulanate	6 (0.7)	0.68
Piperacillin-tazobactam	6 (0.7)	1.71
Ampicillin	1 (0.1)	0.03
Cloxacillin	2 (0.2)	0.26
Fluoroquinolones (Ciprofloxacin)	8 (0.9)	0.61
Macrolides (Azithromycin)	8 (0.9)	0.56
Lincosamides (Clindamycin)	6 (0.7)	0.86
Glycopeptides		
Vancomycin	43 (4.8)	6.30
Teicoplanin	2 (0.2)	0.41
Metronidazole	13 (1.5)	1.07
Polymyxins (Colistin)	3 (0.3)	0.92
Carbapenems (Meropenem)	7 (0.8)	1.40
Total		107.91

Abbreviations: DDD, Defined Daily Dose

The use of antibiotics was documented using the WHO AWaRe policy (16). A total of 17 antibiotics were used during the study period, seven of them belonged to the Access list (Cefazolin, Gentamicin, Amoxicillin, Ampicillin, Cloxacillin, Clindamycin, Metronidazole), nine belonged to the Watch list (Cefuroxime, Ceftriaxone, Ceftazidime, Piperacillin-Tazobactam, Ciprofloxacin, Azithromycin, Vancomycin, Teicoplanin, Meropenem), and only one belonged to the Reserve list (Colistin).

**Fig. 1:**
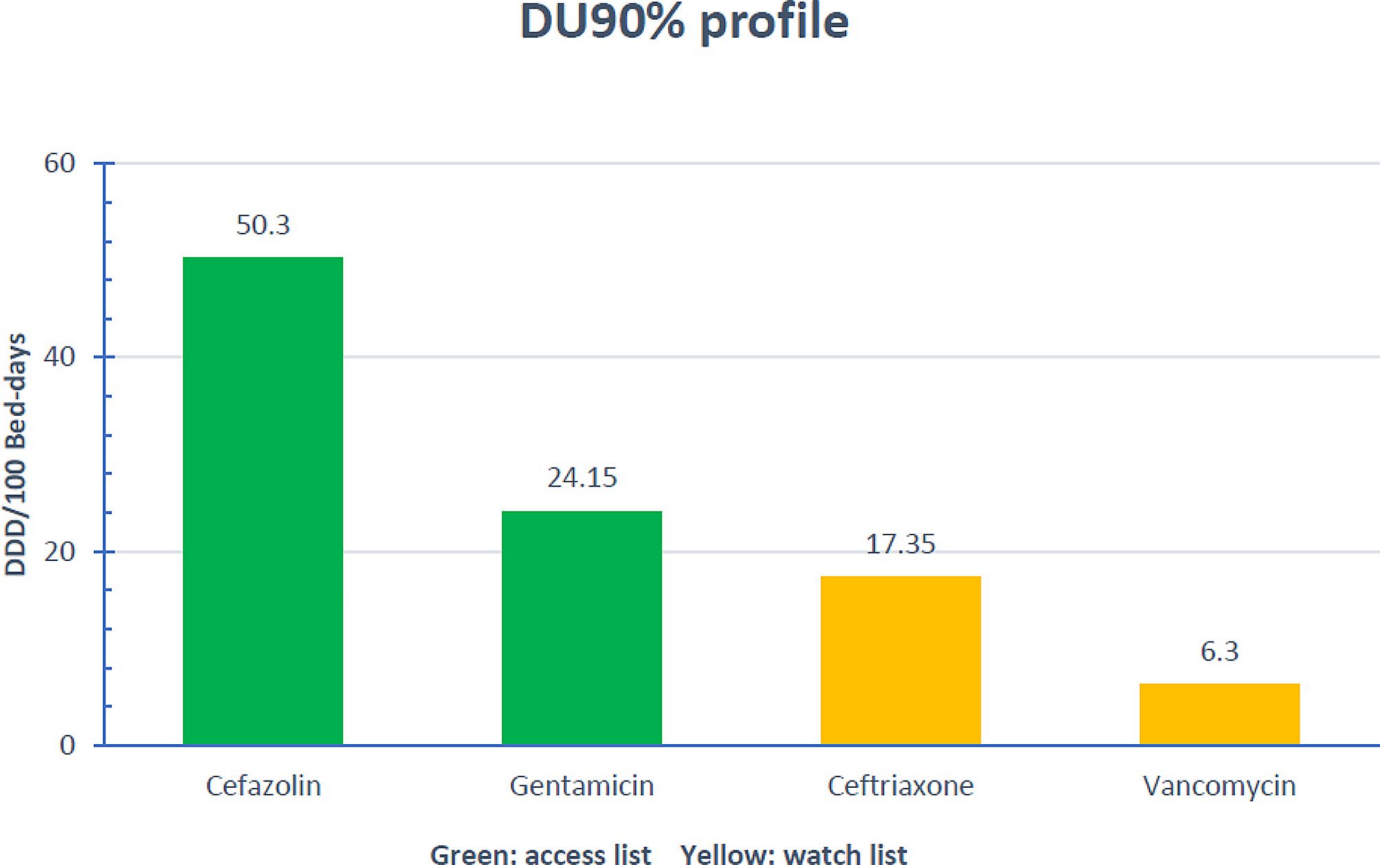
Drug utilization 90% (DU90%) profile of antibiotics according to the WHO AWaRe classification Abbreviations: DDD, Defined Daily Dose

Within the DU90% segment, there were four different agents; two of them (cefazolin and gentamicin) are from the access list and account for 75.9% of antibiotic use in the DU90% segment, the other two (ceftriaxone and vancomycin) are from the watch list. No reserve antibiotics were observed in the DU90%, as shown in Fig. 1.

### Comparison with other hospitals from different regions of the world

Table 4 presents our results and compares it with different areas and hospital around the world. it shows wide variations of the total consumption with different regions around the world. The highest consumption was found in Turkey and Serbia, totaling 173.65 and 173.52 DDD/100 bed days, respectively. Shanghai had the lowest levels of consumption (22.3 DDD/100 bed days), followed by India (23.02 DDD/100 bed days).

**Table 4:** DDD/100 bed days value of antibiotics in orthopedic units from several countries

Country, City/Region	Total DDD/100 bed days of antibiotics consumption in the orthopedic department
Turkey, South-East Anatolian Region (2013) [15]	173.65
Serbia, Nis (2007) [16]	173.52
Germany, Magdeburg (2017) [17]	127.3
Nepal, Biratnagar (2020) [18]	109.33
Palestine, Nablus (2014) [19]	97.75
Iran, Tabriz (2016) [20]	90.17
Indonesia, Surbaya (2022) [21]	74.18
Italy, Emilia-Romagna Region (2014) [22]	64.05
China, Nanjing (2016) [23]	40 + 7
China, Fuzhou (2018) [24] India, Pune (2022) [25]	27.71 23.02
China, Shanghai (2016) [26]	22.30
Our results	107.91

## Discussion

The aim of this study was to measure the defined daily dose per 100 bed days for the orthopedic department in our hospital and to compare it with that of other hospitals worldwide. We found that the total DDD/100 bed days was 107.91. In comparison with other results obtained in studies worldwide, our results are considered high as seen in Table 4.

Compared to our results, studies conducted in certain countries like China found antibiotic consumption to be lower than that in Palestine and many other countries. This can be attributed to the presence of special mandates that restricts the use of antibiotics, as in the case the of China’s rigorous adherence to the National Program of Special Renovation Activity on the Clinical Application of Antibiotics. This initiative was issued by the National Health and Family Planning Commission (NHFPC) in China in 2012, and it mandates that public hospitals maintain their antibiotic consumption below 40 DDD/100 patient-days [24].

Other studies have focused on applying antimicrobial stewardship programs (ASPs) and measuring their effects on total antibiotic consumption [26, 27]. A noteworthy example comes from another study conducted in China, where antibiotic consumption was measured before and after implementing an ASP. This program focused on formulating strategies for rational antibiotic use, through limiting antibiotic options and setting a target of prescribing less than 40 DDD/100 bed days in hospitalized patients and prophylactic antibiotics in clean surgeries. As a result, antibiotic consumption plummeted from 69.23 to 22.30 DDD/100 bed days [26]. Interventions such as ASP can reduce the quantity of antibiotics, which allows for more rational antibiotic use. Interestingly, the application of ASP also decreased the incidence of infections caused by multidrug-resistant organisms (MDROs) infections, as well as the incidence of methicillin-resistant *Staphylococcus aureus* (MRSA) [28]. A study performed in Italy showed that the implementation of an ASP program resulted in decreased hospital length of stay, decreased *Clostridium difficile* colitis incidence, and decreased antimicrobial costs. These studies support the need to implement ASPs in hospitals with high antibiotic consumption.

Regarding the types of antibiotics most used, the most commonly used ones in our study were cefazolin (50.30 DDD/100 bed days), followed by gentamicin (24.15 DDD/100 bed days) and ceftriaxone (17.35 DDD/100 bed days). Our results are similar to those of a study in Iran in which the most commonly used antibiotics were cefazolin (48.8 DDD/100 bed days) and gentamicin (20.26 DDD/100 bed days) [20]. Cefazolin and ceftriaxone are both cephalosporins; which are broad-spectrum antibiotics that can be used against gram-positive and gram-negative infections, including *Staphylococcus aureus* and *Staphylococcus epidermidis*, which are commonly associated with infections in orthopedic surgery [29]. The specific use of cefazolin in surgical prophylaxis aligns with the recommendations of the American Society of Health-System Pharmacists (ASHP) [30]. Additionally, gentamicin in combination with cefazolin was the second most common regimen according to the ASHP guidelines.

In other countries, the most common used antibiotics were different. For example, in Nepal, the most commonly used antibiotics were third-generation cephalosporins. The DDD/100 bed days values for cefixime and ceftriaxone were 27.19 and 22.40, respectively, with a total consumption of 109.33 DDD/100 bed days [18]. On the other hand, the relative high use of ceftriaxone is close to what is found in our study (17.4 DDD/100 bed days). The ASHP and the Scottish Intercollegiate Guidelines Network (SIGN) do not recommended the use of broad-spectrum antibiotics such as ceftriaxone, due to their higher cost and potential to promote resistance, and because they might lead to an increase in the *Clostridium difficile* population [30, 31]. The difference in the prescription patterns of antibiotics might stem from differences in hospital and patient characteristics, hospital antibiotic prescription policies, doctor preferences, or differences in healthcare systems [32].

While there has been an increase in the consumption of antibiotics in the neighboring Arab countries [33, 34], we could not find any studies measuring antibiotic utilization in orthopedic departments using the ATC/DDD methodology. Nonetheless, there have been some studies measuring antibiotic consumption and utilization in Palestinian hospitals using the ATC/DDD methodology [19, 35]. In a study conducted in 2012, researchers collected data for 2 months from inpatients in all departments at the same hospital where we conducted our study. The DDD/100 bed days was 79.75 in the orthopedic department and the most commonly used antibiotic was cefuroxime with a calculated value of 35.02 DDD/100 bed days [19]. Although both studies were conducted at the same hospital, the total consumption of antibiotics in our study was found to be higher. This difference could be due to the longer study period in our case. Additionally, the switch from cefuroxime to cefazolin as the first line prophylactic agent could have played a role in the increased total antibiotic consumption, given that a higher dose of cefazolin is usually given when used as prophylactic compared to cefuroxime.

Regarding the quality of antibiotic prescription, there were four agents in the DU90% segment. The DU90% segment assumes that utilizing fewer agents corresponds to improved quality in prescription patterns. Compared with other countries, our numbers are lower than the DU90% profiles seen in India (11–13 agents) [25, 36], and similar to those in Ethiopia (6–8 agents) [37]. Furthermore, according to the WHO AWaRe policy [11], more than 60% of the total antibiotics used should be from the access list of antibiotics. In our study, antibiotics from the access list accounted for 75.9% of the DU90% antibiotics, while the watch list antibiotics accounted for 24.1%.

Based on the findings of our research study, policy-makers could benefit from recommendations aimed at improving antibiotics utilization and safe use. First, policy-makers could develop and implement ASPs, and set targets for antibiotic consumption at hospitals similar to the effective China’s NHFPC’s program that mandates hospitals not to exceed 40 DDD/100 patient-days of antibiotics consumption [23]. Second, policy-makers are encouraged to standardizing surgical prophylaxis protocols that aligns with international guidelines, as well as regular monitoring of compliance within surgical departments can improve safe and responsible use of antibiotics. Finally, policy-makers could also benefit from enhancing surveillance and reporting systems, educating different healthcare providers, initiating public campaigns to raise awareness, and supporting relevant local and international research to provide evidence-driven data to guide policymaking.

### Limitations and strengths of the study

One of the primary strengths of this study is the large number of patients overall, as it allowed for a diverse range of indications and infections to be included in the study.

Our study had few limitations. First, our study was limited by its single-hospital and single-department focus, thus constraining the generalizability of the results to the whole country. However, this limitation was mitigated with efforts to include a diverse variety of patients with different diagnoses, infections, and indications of antibiotics. Second, the study’s retrospective design might affect the comprehensiveness and depth of our analysis, as it relies on clinical recorded data that was not collected for the purpose of scientific research. However, the use of ATC/DDD methodology as well-trusted and standardized tool ensured the collection of high quality and comparable data that could be analyzed across different times and settings. Moreover, efforts to collect comprehensive and accurate primary data sources was done, and crosschecking from multiple sources and records were followed to minimize the chance of errors.

## Conclusion

Our study is a continuation of previous efforts to combat the growing problem of antibiotics resistance in Palestine. Our results showed that consumption of antibacterial agents in the orthopedic unit at a large governmental hospital in Palestine was relatively high. The results of this study can be helpful to the decision-makers in the country who are responsible for making policies to regulate antibiotic prescriptions. Recommendations to improve the safe use of antibiotics encompass the introduction of ASPs, the standardization of surgical prophylaxis protocols, the enhancement of surveillance systems, the education of healthcare providers, the promotion of public awareness, and the support of local and international relevant research initiatives.

## Data Availability

The datasets used and/or analyzed during the current study are available from the corresponding author upon reasonable request.
